# Dragonfly biodiversity 90 years ago in an Alpine region: The Odonata historical collection of the MUSE (Trento, Italy)

**DOI:** 10.3897/BDJ.7.e32391

**Published:** 2019-01-31

**Authors:** Giacomo Assandri, Alessandra Franceschini, Valeria Lencioni

**Affiliations:** 1 MUSE - Science Museum of Trento, Trento, Italy MUSE - Science Museum of Trento Trento Italy

**Keywords:** dragonflies; damselflies; Italy; natural science museum, Trentino-Alto Adige

## Abstract

**Background:**

Historical collections of natural science museums play a fundamental role in documenting environmental changes and patterns of biodiversity transformation. This considered, they should have a pivotal role to plan conservation and management actions.

The MUSE - Science Museum of Trento is an Italian regional museum preserving about 5.5 million items (organised in 297 collections). About one million of them are invertebrates, 70% of which are of local origin, gathered in the collection "Miscellanea Invertebrati". Odonata account for a minor part of this collection; however, most of them are of local or regional relevance. A complete catalogue of this collection does not exist to date.

**New information:**

The collection was studied in 2017-2018 and this contribution aims to present the Catalogue of the historic collection of Odonata of the MUSE - Museo delle Scienze of Trento (Italy).

In all, 836 specimens of adult dragonflies and damselflies are found in the collection referring to an overall 56 species. The collection covers a period between 1924 and 1957 and refer to 74 defined localities, all located in northern Italy (most of them in Trentino - Alto Adige Region).

The samples conserved in the collection are, for several species, the only indisputable confirmation of their former occurrence in that region.

## Introduction

The MUSE - Science Museum of Trento (formerly Museo Tridentino di Scienze Naturali) is an Italian regional museum founded in 1922. The natural history and archaeological collections of the museum (297 collections and 5.5 million objects) are of great interest for their close relationship with the Alpine Region. The oldest materials were collected more than two centuries ago and the collections increase every year through many new acquisitions. Invertebrate collections of the MUSE include more than 1.2 million specimens, aquatic and terrestrial, 70% of which are of local origin. Historical collections (1850-1950) include mainly terrestrial insects.

Odonata account for a minor part of the "Miscellanea Invertebrati" collection (cINV017), with 954 specimens collected since 1924, mostly in Trentino (NE-Italy). The bulk of the collection (836 specimens) was created between 1924 and 1957 and is referred hereafter as the 'historical collection of dragonflies of the Science Museum (MUSE) of Trento'. The remaining samples refer to 2009 and were collected at two sites of Trentino in the framework of a specific project, the results from which have already been reported in Lampo et al. (2011). Hence, this contribution intends to present the catalogue of the historical bulk of the collection.

The dragonflies in the Museo Tridentino di Scienze Naturali collection were previously studied by Cesare Nielsen in 1932, who reported the available records at that time (which were a small number compared to the ones available today) (Nielsen 1932). They were subsequently studied by Cesare Conci and Osvaldo Galvagni in 1944, who only reported several relevant data for an individual species, *Sympecma paedisca* (Brauer, 1882) (Conci and Galvagni 1944).

This considered, we think it important to publish the full catalogue of the historical collection of dragonflies of the Science Museum of Trento, since these data represent the first organic and verifiable bulk of knowledge on the Odonata of Trentino. In fact, apart from two 19^th^ century very general and not verifiable studies (Ambrosi 1851, Ausserer 1869), no other previous information exists which allow the delineation of the past odonate fauna of this area. Additionally, during the 34 years to which the records in this collection refer, few data on the Odonata of Trentino were published and most of them referred only to scarce species (i.e. apart from those already cited: Morton 1926, Conci and Galvagni 1946, Conci 1957, Conci 1948, Morton 1928, Marcuzzi 1948).

Historical data from the Natural Science Museum collections allow comparisons with present animal assemblages and enable the understanding of the dynamics of the communities (i.e. species extinctions and colonisations) and concurrently of ecosystems (Regneire et al. 2015). These modifications could be the result of natural processes or, more often, of the anthropogenic impacts on biodiversity; thus, collection data play a fundamental role in documenting environmental changes (Schmitt et al. 2018). This also explains their invaluable importance in planning conservation and management actions (Remsen 1995, Gobbi et al. 2012). As an example, of the 61 odonate species recorded in Trentino, four (*Sympecma paedisca*, *Lestes barbarus*, *Coenagrion scitulum* and *Brachytron pratense*) were not reported after 1950 (Assandri, unpublished data). For all of them, at least one sample is conserved in the historical collection of dragonflies of the MUSE, confirming their indisputable former occurrence in that region.

## Sampling methods

### Study extent

The historical specimens of Odonata conserved in the collection "Miscellanea Invertebrati" of MUSE are 836 referring to 74 localities. Specimens are contained in a total of 44 entomological boxes.

### Sampling description

No data on sampling protocols used in the past were available, although it is likely that most of the specimens derived from opportunistic sampling performed by personnel of the Museum, in particular by Guido Castelli and Tullio Perini, in Trentino Alto-Adige. Few specimens come from donations by other entomologists.

The collection is kept dry, most of the specimens are pinned (N=737), whereas the others are conserved in dragonfly envelopes (N=99).

### Quality control

The collection was studied by GA in 2017-2018. All the samples were revised and reordered. A point of strength of the collection is that labels are mostly conserved and complete, thus relevant data about date and locality are available. These were digitalised. Geographical data on labels were georeferenced based on locality names. In most cases, the localities were well defined and straightforward for georeferencing as they referred to specific physical elements (lakes, mountains, wetlands). When the locality referred to a town or a city, we associated it with the approximate present centroid of the urban area, although it could have been more vague (e.g. referred to the municipality). When the information is too imprecise for georeferencing (e.g. valleys) or unclear, we do not provide coordinates.

Taxonomy and nomenclature in this paper and associated dataset follow Boudot and Kalkman (2015).

## Geographic coverage

### Description

**Figure 1.**
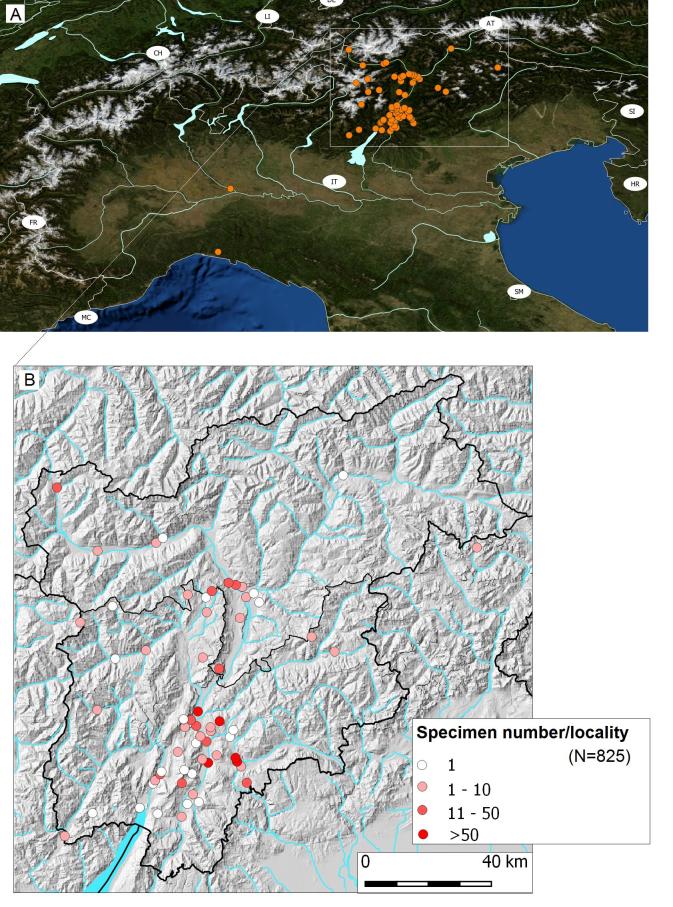
Geographical distribution of the Odonata specimens conserved in the MUSE "Miscellanea invertebrati" collection. **A.** All specimens. Base-map: Northern Italy - USGS The National Map: Orthoimagery. Data refreshed October 2017; **B.** Focus on Trentino - Alto Adige region with number of specimens per locality detailed (N=825).

All the Odonata specimens, deposited in the collection "Miscellanea Invertebrati" of MUSE, geographically refer to Northern Italy. Most of them come Central-Eastern Alps, specifically from Trentino (N=692) and Alto Adige (N=138). Another 3 specimens come from Veneto (all 3 *Leucorrhinnia dubia*), 1 from Lombardia (*Calopteryx splendens*) and 2 from Liguria (*Calopteryx xanthostoma*) (Fig. 1A). Overall, data for 74 localities are available (collection effort per locality: 1-122 Fig. 1B).

**Figure 2.**
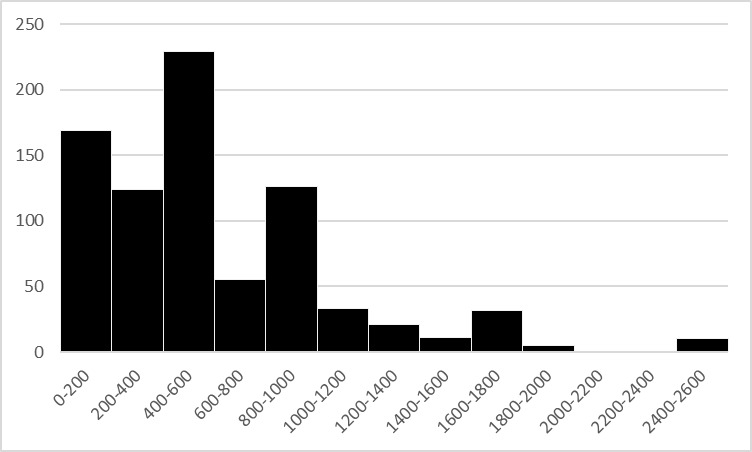
Altitudinal distribution of Odonata specimens in the MUSE "Miscellanea invertebrati" collection (N=815).

Specimens refer to an altitudinal gradient between 66 and 2600 m a.s.l., although the 86% of them were collected at low elevation (within 1000 m a.s.l.) (Fig. 2). Considering that most of the data came from a region which extends for 70% above 1000 m a.s.l. (Rossi 2005), this evidence suggests a possible disproportionate sampling tendency towards the valley bottom, while admitting that the diversity of dragonflies in the Alps is concentrated at lower altitudes.

### Coordinates

44.449 and 46.815 Latitude; 12.265 and 9.012 Longitude.

## Taxonomic coverage

### Description

**Figure 3.**
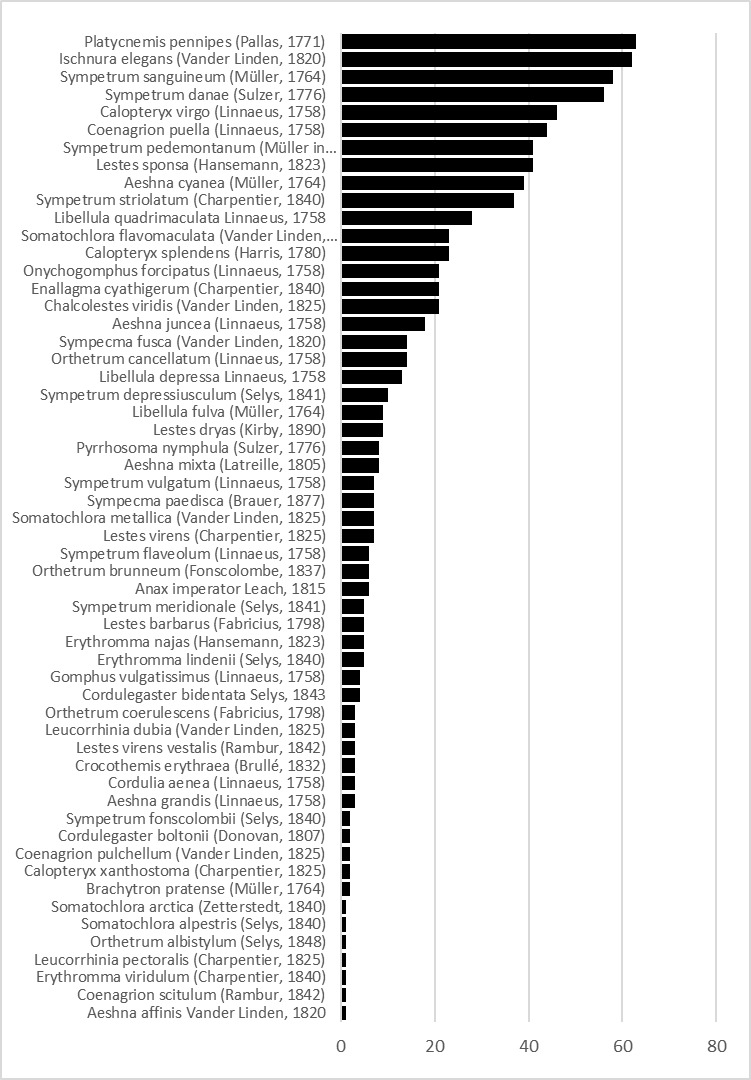
Number of Odonata specimens in the MUSE "Miscellanea invertebrati" collection divided by species (N=836).

A total of 56 Odonata species are represented in the MUSE "Miscellanea Invertebrati" collection (Fig. 3). Those represent 59% of the 95 species recorded at least once in Italy (http://www.odonata.it/libe-italiane) and 39% of the 143 species recorded in Europe (Boudot and Kalkman 2015).

### Taxa included

**Table:** 

Rank	Scientific Name	
kingdom	Animalia	
phylum	Artropoda	
class	Insecta	
order	Odonata	
species	*Aeshna affinis* Vander Linden, 1820	
species	*Aeshna cyanea* (Müller, 1764)	
species	*Aeshna grandis* (Linnaeus, 1758)	
species	*Aeshna juncea* (Linnaeus, 1758)	
species	*Aeshna mixta* (Latreille, 1805)	
species	*Anax imperator* Leach, 1815	
species	*Brachytron pratense* (Müller, 1764)	
species	*Calopteryx splendens* (Harris, 1780)	
species	*Calopteryx virgo* (Linnaeus, 1758)	
species	*Calopteryx xanthostoma* (Charpentier, 1825)	
species	*Chalcolestes viridis* (Vander Linden, 1825)	
species	*Coenagrion puella* (Linnaeus, 1758)	
species	*Coenagrion pulchellum* (Vander Linden, 1825)	
species	*Coenagrion scitulum* (Rambur, 1842)	
species	*Cordulegaster bidentata* Selys, 1843	
species	*Cordulegaster boltonii* (Donovan, 1807)	
species	*Cordulia aenea* (Linnaeus, 1758)	
species	*Crocothemis erythraea* (Brullé, 1832)	
species	*Enallagma cyathigerum* (Charpentier, 1840)	
species	*Erythromma lindenii* (Selys, 1840)	
species	*Erythromma najas* (Hansemann, 1823)	
species	*Erythromma viridulum* (Charpentier, 1840)	
species	*Gomphus vulgatissimus* (Linnaeus, 1758)	
species	*Ischnura elegans* (Vander Linden, 1820)	
species	*Lestes barbarus* (Fabricius, 1798)	
species	*Lestes virens* (Charpentier, 1825)	
species	*Lestes dryas* (Kirby, 1890)	
species	*Lestes sponsa* (Hansemann, 1823)	
species	*Lestes virens* (Charpentier, 1825)	
species	*Leucorrhinia dubia* (Vander Linden, 1825)	
species	*Leucorrhinia pectoralis* (Charpentier, 1825)	
species	*Libellula depressa* Linnaeus, 1758	
species	*Libellula fulva* (Müller, 1764)	
species	*Libellula quadrimaculata* Linnaeus, 1758	
species	*Onychogomphus forcipatus* (Linnaeus, 1758)	
species	*Orthetrum albistylum* (Selys, 1848)	
species	*Orthetrum brunneum* (Fonscolombe, 1837)	
species	*Orthetrum cancellatum* (Linnaeus, 1758)	
species	*Orthetrum coerulescens* (Fabricius, 1798)	
species	*Platycnemis pennipes* (Pallas, 1771)	
species	*Pyrrhosoma nymphula* (Sulzer, 1776)	
species	*Somatochlora alpestris* (Selys, 1840)	
species	*Somatochlora arctica* (Zetterstedt, 1840)	
species	*Somatochlora flavomaculata* (Vander Linden, 1825)	
species	*Somatochlora metallica* (Vander Linden, 1825)	
species	*Sympecma fusca* (Vander Linden, 1820)	
species	*Sympecma paedisca* (Brauer, 1877)	
species	*Sympetrum danae* (Sulzer, 1776)	
species	*Sympetrum depressiusculum* (Selys, 1841)	
species	*Sympetrum flaveolum* (Linnaeus, 1758)	
species	*Sympetrum fonscolombii* (Selys, 1840)	
species	*Sympetrum meridionale* (Selys, 1841)	
species	*Sympetrum pedemontanum* (Müller in Allioni, 1766)	
species	*Sympetrum sanguineum* (Müller, 1764)	
species	*Sympetrum striolatum* (Charpentier, 1840)	
species	*Sympetrum vulgatum* (Linnaeus, 1758)	

## Traits coverage

### Data coverage of traits

PLEASE FILL IN TRAIT INFORMATION HERE

## Temporal coverage

### Notes

**Figure 4.**
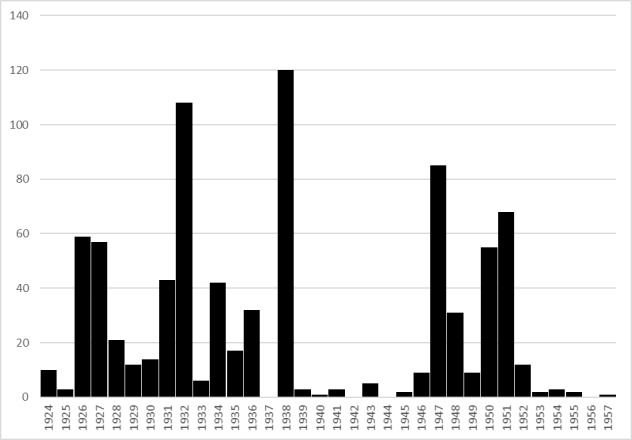
Temporal distribution of Odonata specimens in the MUSE "Miscellanea invertebrati" collection (N=835).

The Odonata specimens deposited in the MUSE "Miscellanea invertebrati collection" cover a timespan of 34 years between 1924 and 1957 (Fig. 4). This motivated the name ("historical") chosen to designate this collection as an unique entity, which in fact is the result of heterogeneous entomological activities carried on by different collectors. It is noteworthy to mention the almost total cessation of the collecting activities during the years of World War II (1940-1945).

## Collection data

### Collection name

"Miscellanea invertebrati" - MUSE

### Collection identifier

cINV017

### Parent collection identifier

MUSE

### Specimen preservation method

Dried specimens (pinned; dragonfly envelopes).

## Usage rights

### Use license

Creative Commons Public Domain Waiver (CC-Zero)

### IP rights notes

This work is licensed under a Creative Commons Attribution (CC-BY) 4.0 License.

## Data resources

### Data package title

Historical collection of dragonflies (Insecta : Odonata) of the Science Museum (MUSE) of Trento

### Resource link

http://ipt.pensoft.net/resource?r=muse_odonata

### Number of data sets

1

### Data set 1.

#### Data set name

Historical collection of dragonflies (Insecta : Odonata) of the Science Museum (MUSE) of Trento

#### Data format

Darwin Core

#### Number of columns

41

#### 

**Data set 1.** 

Column label	Column description
type	The nature of the resource
language	The language of the resource
institutionCode	The name in use by the institution having custody of the object or information referred to in the record
collectionCode	The name and acronym identifying the collection from which the record was derived
datasetName	The name identifying the dataset from which the record was derived
basisOfRecord	The specific nature of the data record
dynamicProperties	box: the entomological box number in which the specimen is conserved
catalogNumber	An unique identifier for the record within the dataset and collection
occurrenceRemarks	Notes about the Occurrence
recordedBy	A person responsible for recording the original Occurrence (legit)
individualCount	The number of specimen available for an Occurrence
sex	The sex of the specimen represented in the Occurrence
lifeStage	The age class or life stage of the specimen of the Occurrence
preparations	Preparations and preservation methods for the specimen
eventDate	Date when the specimen was collected (according to label)
year	The four-digit year in which the Event occurred, according to the Common Era Calendar
month	The ordinal month in which the Event occurred
day	The integer day of the month on which the Event occurred
continent	The name of the continent in which the Location occurs
country	The name of the country in which the Location occurs
stateProvince	The name of the next smaller administrative region than country (region) in which the Location occurs
county	The name of the next smaller administrative region than stateProvince (Province) in which the Location occurs
municipality	The full, unabbreviated name of the next smaller administrative region than county (municipality) in which the Location occurs
locality	The specific description of the place. This term may contain information modified from the original to correct perceived errors or to standardise the description.
verbatimLocality	The original textual description of the place
verbatimElevation	The original description of the elevation (altitude, usually above sea level) of the Location
minimumElevationInMeters	The lower limit of the range of elevation (altitude, usually above sea level), in metres. This is referred to georeferenced Location
decimalLatitude	The geographic latitude (in decimal degrees, using the spatial reference system given in geodeticDatum) of the geographic centre of a Location
decimalLongitude	The geographic longitude (in decimal degrees, using the spatial reference system given in geodeticDatum) of the geographic centre of a Location
geodeticDatum	The geodetic datum upon which the geographic coordinates given in decimalLatitude and decimalLongitude as based
georeferencedBy	Who determined the georeference (spatial representation) for the Location
georeferenceVerificationStatus	A categorical description of the extent to which the georeference has been verified to represent the best possible spatial description
identifiedBy	A list (concatenated and separated) of names of people who assigned the Taxon to the subject
scientificName	The full scientific name, with authorship and date information
order	The full scientific name of the order in which the taxon is classified
taxonRank	The taxonomic rank of the most specific name in the scientificName
occurrenceID	A globally unique identifier for the Occurrence
genus	The full scientific name of the genus in which the taxon is classified
specificEpithet	The name of the first or species epithet of the scientificName
infraspecificEpithet	The name of the lowest or terminal infraspecific epithet of the scientificName, excluding any rank designation
scientificNameAuthorship	The authorship information for the scientificName formatted according to the conventions of the applicable nomenclaturalCode

## Additional information

Assandri G (2018): Dragonfly biodiversity 90 years ago in an Alpine region: The Odonata historical collection of the MUSE (Trento, Italy). v1.6. Biodiversity Data Journal. Dataset/Occurrence. http://ipt.pensoft.net/resource?r=muse_odonata
